# Irisin as a predictor of bone metabolism in Han Chinese Young Men with pre-diabetic individuals

**DOI:** 10.1186/s12902-022-01199-w

**Published:** 2022-11-18

**Authors:** Junru Liu, Xing Wang, Dongmei Fan, Lina Sun, Weinan Zhang, Fuzai Yin, Bowei Liu

**Affiliations:** Department of Endocrinology, The First Hospital of Qinhuangdao Hebei Medical University, No.258 Wenhua Road, 066000 Qinhuangdao, Hebei Province China

**Keywords:** Irisin, β-C-telopeptides of type I collagen (CTX), Procollagen 1 amino-terminal propeptide (P1NP), Osteocalcin(OC), Body mass index (BMI), Impaired glucose regulation (IGR)

## Abstract

**Background:**

Irisin is a novel myokine both in mice and humans, and it can also be secreted by adipose tissue and the liver in a small amounts. There are few studies on irisin and bone metabolism. The aim of this study was to assess the relationship between serum irisin levels and bone metabolism and analyze its related factors in Han young male with pre-diabetic individuals.

**Methods:**

This cross-sectional study included 41 pre-diabetes and 45 normal glucose tolerance (NGT). Anthropometric measurements, including height, weight, waist circumference (WC), and bone mineral content (BMC), were performed. All patients underwent an oral glucose tolerance test (OGTT) after 8 h of fasting, and the levels of glucose, insulin, lipids, serum irisin and bone turnover markers were measured.

**Results:**

The levels of serum irisin (4.4 ± 1.4 vs. 6.3 ± 1.5 µg/mL), P1NP and OC were significantly lower and CTX was significantly higher in the pre-diabetes group (*P* < 0.05). BMC did not differ in the two groups (*P* > 0.05). Serum irisin levels negatively correlated with BMI (*r* =-0.325), FPG (*r* =-0.329), TG (*r* =-0.339) (*P* < 0.05) in NGT individuals. Serum irisin levels positively correlated with P1NP (*r* = 0.398), OC (*r* = 0.351), HDL-C (*r* = 0.432) and negatively correlated with FPG (*r* = -0.725), 2 h-PG (*r* = -0.360) (*P* < 0.05) in pre-diabetic individuals. Multiple regression analysis revealed that Serum irisin (*β* = 9.768, *P* = 0.025) and WC (*β* = -2.355, *P* = 0.002) were significant independent predictors for P1NP.

**Conclusion:**

Bone turnover markers were changed rather than bone mineral content in young men with pre-diabetes. In pre-diabetes individuals, serum irisin levels were reduced and close relationship with P1NP. Falling irisin levels may be a predictor of decreased bone formation in Han young men with pre-diabetes individuals.

## Introduction

Irisin is a protein consisting of 112 amino acids, and obtained by cleavage of its precursor fibronectin type III domain-containing (FNDC5). It is an exercise-induced myokine possibly leading to the browning of white adipose tissue, thereby increasing energy expenditure and improving systemic metabolism [1]. Most available evidence shows that irisin significantly influences glucose and energy homeostasis [2–4]. Circulating irisin was found to be significantly reduced in long-term [5], new onset [6], and undefined [7] T2DM patients compared with nondiabetic controls, which suggested either the diabetic state itself or the metabolic condition that caused progression to T2DM is accompanied by lower circulating irisin [8, 9].

People with type 2 pre-diabetes also have the basis for the occurrence of macrovascular disease. People in this stage are characterized by more prominent factors such as insulin resistance and obesity [10], and obesity and insulin resistance are inextricably linked with bone metabolism. However, besides energy expenditure, exercise also has beneficial on the bone metabolism. Bone is a metabolically dynamic tissue consisting of osteoblasts, osteocytes, and osteoclasts [11]. It is well known that bone metabolism can be regulated by a range of factors, including physical exercise. Physical exercise acts on the bone directly via mechanical force, or indirectly via an anabolic effect through hormonal factors [12]. Recently, irisin has received attention from investigators who study osteoporosis and bone metabolism. It has been well demonstrated that skeletal muscle and bone structure are closely correlated, and many studies reporting correlations between lean mass, bone mass, and bone fracture risk can be found in the literature [13, 14]. The study represented in elderly population that the positive correlation between plasma irisin and bone mineral density (BMD) hints intrinsic communication between muscle and bone [15, 16]. Literature showed that the association of highest irisin amounts to a better glycaemic control and bone health in T1DM subjects on CSII [17]. Serum irisin levels are positively correlated with bone mineral status in a population of healthy children [18].

Although it has been recently studied for the relationship of BMD and plasma irisin in children of T1DM and elderly populations, it is still unknown whether plasma irisin is related to bone metabolism in young adults with peak bone mass without diabetes. We therefore adopted a design to evaluate the relationship between serum irisin levels and bone metabolism and analyze its related factors in Han young male with pre-diabetic individuals.

## Materials and methods

### Study design

We performed a cross-sectional study in Chinese young male with nondiabetic and pre-diabetic individuals. After the informed consent was obtained from all subjects, 86 adult men (aged 20 to 45 years) participated in the present study. All subjects were of the Han ethnicity, and each underwent an oral glucose tolerance test (OGTT) with 75 g of oral anhydrous glucose. The inclusion criteria were: (1) 18–45 year old; (2) absence of pregnancy or breast-feeding, (3) stable body weight (bodyweight change < 2 kg over the 3 months before enrollment), (4) daily light physical activity, and (5) willingness to take part in the examination and willingness to provide blood samples. The exclusion criteria included the following: (1) with diseases that may cause osteoporosis: hypercortisol, hyperthyroidism, hyperparathyroidism, rheumatoid arthritis or other endocrine diseases, autoimmune diseases; (2) with taking drugs affecting bone metabolism recently; 3)with gastrointestinal diseases and acute infections; 4) with moderate or severe liver and kidney damage; 5) heavy smokers (of smoking more than 10 cigarettes a day and for more than 10 years), coffee drinkers and heavy drinkers; 6) stay in bed for a long time. This study was approved by the ethics committee of the Qinhuangdao First hospital.

### Cases and definition

We enrolled 41 pre-diabetes and 45 normal glucose tolerance(NGT) with who had gone to the First Hospital of Qinhuangdao for health examinations during 2014 to 2015. According to 2008 American Diabetes Association diabetes diagnostic criteria, NGT was defined as fasting plasma glucose (FPG) levels that were < 5.6 mmol/L and 2-h plasma glucose (2-h PG) levels that were < 7.8 mmol/L after a 75-g OGTT. Impaired glucose regulation(IGR) was defined as FPG levels that were ≥ 5.6 mmol/L but < 7.0 mmol/L and/or 2-h PG levels that were ≥ 7.8 mmol/L but < 11.1 mmol/L after a 75-g OGTT.

### Anthropometric measurements

Anthropometric measurements, including height, weight, waist circumference and bone mineral content (BMC) were obtained while the subjects were in light clothing and not wearing shoes. Body mass index (BMI) was calculated by dividing weight (kg) by height squared (m^2^). Waist circumference (WC) was measured with the subject in a standing position at the midpoint between the lateral iliac crest and the lowest rib at the end of expiration while the subject was breathing gently. BMC(g) of the lumbar spine and femoral neck was detected using dual energy X-ray absorptiometry (DXA) (GE bone density analyzer). The inspection was performed daily by the same professional physician. The coefficient of variation (CV) for repeated measurements was approximately 1.0%.

### Laboratory examinations

All subjects underwent OGTT with 75 g of oral anhydrous glucose at 8:00 AM after 8 h of fasting. 75 g anhydrous glucose was dissolved in 250 mL water. Peripheral venous blood samples were taken at 0 and 120 min after glucose loading. Plasma glucose concentration was measured using the glucose oxidase method and serum lipids were measured using enzymatic procedures with an autoanalyzer (Hitachi, Tokyo, Japan). Serum irisin levels were determined using a commercially available human ELISA kit (Bio Vision, Milpitas, CA 95,035 USA). The sensitivity of the assay was 0.2 µg/mL. The intra and inter-assay variations were both less than 10%. The Osteocalcin ELISA kit was purchased from American abcam (internal variation coefficient was less than 8.54%, inter-assay coefficient of variation was less than 3.73%). CTX ELISA kit was purchased from LifeSpan, USA (the intra-assay coefficient of variation was less than 10%, and the inter-assay coefficient of variation was less than 10%). P1NP ELISA kit was purchased from BIOLOGY, USA (the intra-assay coefficient of variation was 6.3% or less, and the inter-assay coefficient of variation was 8.5% or less).The ELISA kits of insulin were purchased from USCNLIFE company, USA. Insulin, Osteocalcin, CTX, P1NP and serum irisin were measured using an enzyme linked immunosorbent assay (ELISA) with a model 680 microplate reader (BIO-RAD, USA). The following equation for homeostasis model assessment of insulin resistance (HOMA-IR) was used: fasting insulin level (µU/mL) x fasting glucose level (mmol/L)/22.5.

### Statistical analysis

Data are expressed as mean ± standard deviation (SD) or medians with interquartile ranges (IQR). When data was not normally distributed, they were log transformed for analysis. Comparisons were conducted between groups using the t test. To measure the strength of association between 2 variables, Pearson’s correlation coefficient was used. To examine the association between irisin and bone turnover markers, multiple linear regression analysis was used. Analyses were performed with the computer software SPSS version 11.5 software (SPSS Inc., Chicago, IL, U.S.A.). Statistical significance was established at *P* < 0.05.

## Results

**Fig. 1:**
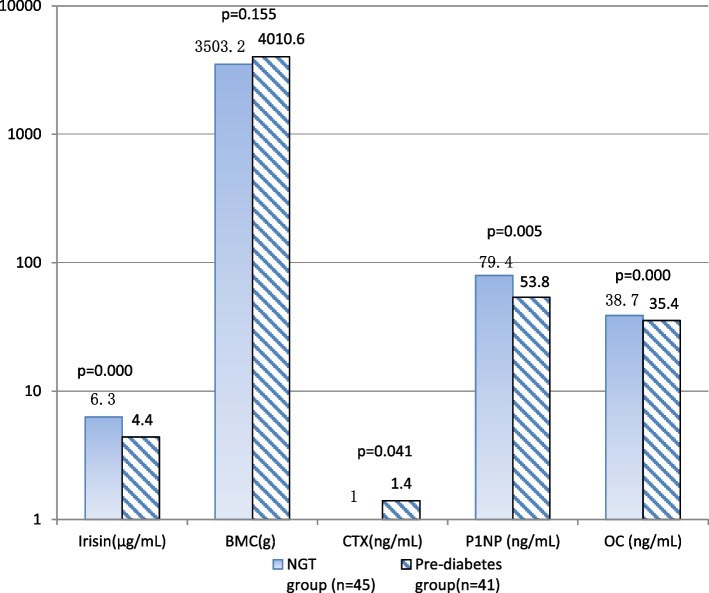
Comparison of irisin, BMC, CTX, OC and P1NP of the subjects between NGT group and pre-diabetes groups

The age, triglycerides (TG), and bone mineral content (BMC) were similar in the two groups (*P* > 0.05). Table 1 showed clinical and laboratory characteristics in the study subjects. Subjects in the pre-diabetes group had higher BMI, WC, FPG, 2-h PG, HOMA-IR, OC, P1NP, and lower CTX, high density lipoprotein cholesterol (HDL-C) than subjects in the NGT group (*P* < 0.05) (Fig. 1). The levels of serum irisin (4.4 ± 1.4 vs. 6.3 ± 1.5 µg/mL) was significantly lower in the pre-diabetes group (*P* < 0.05) (Fig. 1).

**Table 1:** Clinical and laboratory characteristics of the subjects in different groups

variable	NGT group (*n* = 45)	Pre-diabetes group (*n* = 41)	*t* or *χ*^2^	*P*
Age (years) mean (SD)	37.1 (6.8)	37.8 (4.8)	-0.576	0.566
BMI (kg/m^2^) mean (SD)	26.8 (3.5)	29.1 (2.7)	-3.517	0.001
WC (cm) mean (SD)	93.4 (9.1)	97.7 (8.4)	-2.244	0.027
FPG (mmol/L) mean (SD)	5.2 (0.2)	5.8 (0.5)	-7.266	0.000
2 h-PG (mmol/L) mean (SD)	5.8 (0.9)	7.7 (1.9)	-5.743	0.000
TG (mmol/L) median (IQR)	1.24 (0.17)	1.56 (0.36)	-0.606	0.546
HDL-C (mmol/L) mean (SD)	1.38 (0.25)	1.24 (0.33)	3.715	0.000
FINS(uIU/mL) median (IQR)	10.73 (2.6)	11.93 (3.1)	-1.778	0.079
HOMA-IR median (IQR)	2.48 (0.62)	3.29 (1.04)	-2.905	0.005
InIR mean(SD)	0.9 (0.4)	1.2 (0.4)	-2.924	0.004
Irisin (µg/mL) mean (SD)	6.3 (1.5)	4.4 (1.4)	5.804	0.000
BMC (g) mean (SD)	3503.2 (1414.1)	4010.6 (1583.4)	-1.439	0.155
CTX (ng/mL) mean (SD)	1.0 (0.5)	1.4 (1.0)	-2.071	0.041
P1NP (ng/mL) mean (SD)	79.4 (34.8)	53.8 (45.0)	2.924	0.005
OC (ng/mL) mean (SD)	38.7 (3.5)	35.4 (4.4)	3.856	0.000

Data are expressed as mean ± standard deviation (SD) or medians with interquartile ranges (IQR). When data was not normally distributed, they were ln transformed for analysis

*NGT* normal glucose tolerance, *BMI *body mass index, *WC *waist circumference, *FPG *fasting plasma glucose, *2 h-PG *2 h plasma glucose, *TG *triglycerides, *HDL-C *high density lipoprotein cholesterol, *FINS *fasting insulin, *HOMA-IR *homeostasis model assessment of insulin resistance, *BMC *bone mineral content, *CTX *β-C-telopeptides of type I collagen, *P1NP *Procollagen 1 Amino-terminal Propeptide, *OC *Osteocalcin, *SD *standard deviation, *IQR *indicates interquartile range

Table 2 showed that serum irisin levels negatively correlated with BMI (*r* =-0.325), FPG (*r* =-0.329), TG (*r* =-0.339) (*P* < 0.05) in NGT individuals. Serum irisin levels positively correlated with P1NP (*r* = 0.398), OC (*r* = 0.351), HDL-C (*r* = 0.432) and negatively correlated with FPG (*r* = -0.725), 2 h-PG (*r* = -0.360) (*P* < 0.05) in pre-diabetic individuals. Serum irisin levels positively correlated with P1NP (*r* = 0.343). OC (*r* = 0.434), HDL-C (*r* = 0.437) and negatively correlated with BMI (*r* = -0.385), WC (*r* = -0.310), FPG (*r* = -0.699), TG (*r* = -0.185) (*P* < 0.05) in all the study subjects (Table 2).

**Table 2:** Simple correlations between the Irisin and other variables in all subjects, NGT group and Pre-diabetes group in the study

Variable	All subjects		NGT group		Pre-diabetes group	
	*r*	*P*	*r*	*P*	*r*	*P*
CTX (ng/mL)	-0.160	0.140	0.147	0.335	-0.156	0.331
P1NP (ng/mL)	0.343	0.001	0.021	0.890	0.398	0.010
OC (ng/mL)	0.434	0.000	0.225	0.137	0.351	0.025
BMC (g)	-0.064	0.590	-0.008	0.965	0.073	0.665
Age (years)	0.049	0.653	-0.062	0.685	0.061	0.705
BMI (kg/m^2^)	-0.385	0.000	-0.325	0.029	-0.138	0.388
WC (cm)	-0.310	0.004	-0.249	0.099	-0.193	0.227
FPG (mmol/L)	-0.699	0.000	-0.329	0.027	-0.725	0.000
2 h-PG (mmol/L)	-0.145	0.182	-0.051	0.739	-0.360	0.021
TG (mmol/L)	-0.302	0.005	-0.339	0.023	-0.301	0.056
HDL-C (mmol/L)	0.437	0.000	0.173	0.256	0.432	0.005
In HOMA-IR	-0.145	0.183	-0.060	0.697	0.107	0.507

*BMI* body mass index, *WC *waist circumference, *FPG *fasting plasma glucose, *2 h-PG *2 h plasma glucose, *TG *triglycerides, *HDL-C *high density lipoprotein cholesterol, *FINS *fasting insulin, *HOMA-IR *homeostasis model assessment of insulin resistance, *BMC *bone mineral content

In the pre-diabetes group, when CTX, OC and P1NP were considered as the dependent variable respectively in a multiple regression analysis with age, BMI, WC, TG, HDL-C, FPG, and 2-h PG and HOMA-IR as independent variables, WC (*β* = 0.061, *P* = 0.002) was still significantly associated with CTX, FPG (*β* = -3.436, *P* = 0.019) was still significantly associated with OC, WC (*β* = -2.355, *P* = 0.002) and Irisin (*β* = 9.768, *P* = 0.025) were still significantly associated with P1NP (Table 3).

**Table 3:** Multiple linear regression analyses for CTX, OC and P1NP in Pre-diabetes (Stepwise Method)

	Model	Unstandardized Coefficients B	Std. Error	Standardized Coefficients B	*t*	*P*	95% *CI*	*R*^2^
1	(Constant)	-4.569	1.815		-2.518	0.016	-8.239 to -0.899	
	WC	0.061	0.019	0.467	3.294	0.002	0.024 to 0.098	0.218
2	(Constant)	55.187	8.159		6.764	0.000	38.685 to 71.689	
	FPG	-3.436	1.409	-0.364	-2.439	0.019	-6.285 to -0.586	0.132
3	(Constant)	240.681	76.023		3.166	0.003	86.781 to 394.582	
	WC	-2.355	0.717	-0.440	-3.285	0.002	-3.806 to -0.904	0.250
	Irisin	9.768	4.172	0.313	2.342	0.025	1.323 to 18.213	0.345

1. Dependent Variable: CTX; 2. Dependent Variable: OC; 3. Dependent Variable: P1NP

## Discussion

In the present study, we selected young men with peak bone mass as the study subjects, excluding gender and menopause. We found that the serum irisin levels, P1NP, OC were reduced and CTX was elevated in Han young adults with pre-diabetes individuals. However, BMC did not differ in the two groups. In addition, the subjects in the pre-diabetes group had higher BMI, FPG, 2-h PG, HOMA-IR, and lower HDL-C than subjects in the NGT group. Although we excluded the physiological effects of age, gender, and menopause on bone metabolism [19], we still found that serum irisin still plays an important role in bone metabolism. We sought to determine whether serum irisin levels were associated with clinical indicators of bone turnover in nondiabetic individuals.

In the study, there was a negative correlation between serum irisin levels and BMI, FPG and TG, not correlation with bone turnover markers in the NGT individuals. However, serum irisin levels positively correlated with P1NP, OC, HDL-C and negatively correlated with glucose and multiple regression analysis revealed that serum irisin and WC were significant independent predictors for P1NP in the pre-diabetes individuals. In additional, multiple regression analysis showed that WC was significant independent predictors for CTX not only in NGT but also in pre-diabetes individuals. The results of our study indicated that although BMC did not change in young men with pre-diabetes, bone turnover markers had changed, and serum irisin level was closely related to P1NP. Whether or not the young men with abdominal obesity have abnormal glucose metabolism, their bone formation ability decreases.

People with type 2 pre-diabetes are characterized by more prominent factors such as insulin resistance and obesity [10]. Ian J. Neeland [20] found that even among obese individuals with normal FBG levels at enrollment, those who subsequently developed prediabetes or diabetes had baseline evidence of insulin resistance (higher HOMA-IR) and impaired intermediate-term glycemic control (higher fructosamine level), with moderate elevations in HOMA-IR and fructosamine among those who developed prediabetes and more marked elevations in those who developed diabetes. Obesity can cause inflammatoin [21, 22], aggravate insulin resistance, and are also associated with abnormal irisin secretion. And obesity and insulin resistance are inextricably linked with bone metabolism. Over the last few years, irisin has received attention from investigators who study osteoporosis and bone metabolism. Colaianni, et al. [23], observed that mice subjected to 3 weeks of wheel running had increased numbers of murine muscle cells that produced and released higher quantities of irisin than did resting mice. It was followed up that treatment with recombinant irisin (r-irisin) increases cortical bone mass in young healthy mice 2 and prevents bone loss in non-weight-bearing mice [24]. Studies of irisin in relation to bone mineral density (BMD) and fractures have been reported differently at different ages and genders. The literature found in soccer players that irisin levels were positively correlated with total body BMD and with BMD at different bone sites, suggesting a systemic effect of irisin on bone, independently of site-specific mechanical loading. [13]. Yan J et al. reported that low concentrations of irisin in older women were independently associated with increased risk of hip fractures when adjusted for BMD or FRAX score in China [25]. Anastasilakis [26] and Palermo et al. [27], who investigated the relationship between irisin and vertebral fractures in a cross-sectional study that enrolled postmenopausal women with severe osteoporosis. No significant correlation was observed between irisin and BMD at any site, or between irisin and lean mass. The circulating levels of irisin were lower in subjects with previous osteoporotic fractures than in control subjects, even after the irisin levels were adjusted for confounding factors such as creatinine, vertebral and femoral BMD, lean mass, and vitamin D. However, our results were different from the above research. We found that serum irisin levels were strongly associated with PINP and were not associated with BMC in young men without diabetes at peak bone mass. Abdominal obesity may be a factor that leads to decreased bone formation and is not related to glucose metabolism. It implies that irisin may play an important role in bone metabolism. Although most of the human studies that have analyzed interactions between bone health and irisin have several limitations.

Of course the exact mechanism of the association between irisin and bone metabolism remains to be clarified. So far, several possible biological pathways might be proposed to explain the observed findings. Firstly, irisin affects the ability of bone marrow stromal cells to differentiate into mature osteoblasts [23]. Secondly, irisin can induce the differentiation of bone marrow stem cells into osteoblasts, and thereby promote the secretion of osteokines (e.g. osteopontin) which induce new bone development. Irisin performs a loading-mimetic function and might mediate loading-induced increases in osteopontin expression [28]. In conclusion, irisin appears to directly affect the differentiation of osteoblasts into bone cells, and produce an indirect effect mediated by brown adipose tissue. One study showed that irisin promoted osteoclast precursor cell proliferation but inhibited osteoclast differentiation [29]. An another study demonstrates that irisin acts directly on osteoclast progenitors to increase differentiation and promote bone resorption, supporting the tenet that irisin not only stimulates bone remodeling but may also be an important counter-regulatory hormone [30]. In a study by Kim et al., treatment of osteocyte-like (MLO-Y4) cells with physiologically relevant concentrations (1-500 ng/mL) of irisin for 16 h resulted in significantly reduced hydrogen peroxide (H2O2)-induced apoptosis, suggesting that irisin can block osteocyte cell death [31]. The possible role played by physical activity in the interactions between muscle tissue and bone tissue has been the object of intense research ever since the discovery of irisin.

Admittedly, our study had some limitations that deserved to be considered when interpreting the results. First concerns the cross-sectional design, which precludes the establishment of a causation between events. The second, it only included adults of the Han ethnicity, limiting the ability to apply to other ethnic groups. The third was the low number of subjects included on each group that can decrease the power of the statistical analysis performed. The fourth, data on other confounding factors such as muscle mass and physical activity were not considered in this study.

## Conclusion

The present study demonstrates that bone turnover markers were changed rather than bone mineral content in young men with pre-diabetes. In pre-diabetes individuals, serum irisin levels were reduced and close relationship with P1NP. Falling irisin levels may be a predictor of decreased bone formation in Han young men with pre-diabetes individuals and should be examined in future studies.

## Data Availability

The datasets used and/or analyzed during the current study are available from the corresponding author on reasonable request.

## Abbreviations

BMC: bone mineral content
BMD: bone mineral density
BMI: body mass index
CTX: β-C-telopeptides of type I collagen
ELISA: Enzyme linked immunosorbent assay
FNDC5: fibronectin type III domain-containing
FPG: Fasting plasma glucose
HDL-C: High-density lipoprotein cholesterol
IGR: impaired glucose regulation
HOMA-IR: homeostasis model assessment of insulin resistance
NGT: normal glucose tolerance
OC: Osteocalcin
OGTT: oral glucose tolerance test
P1NP: Procollagen 1 Amino-terminal Propeptide
TG: Triglycerides
T2DM: Type 2 diabetes mellitus
2-h PG: 2 h plasma glucose
WC: waist circumference
